# Host-specific differences in the response of cultured macrophages to *Campylobacter jejuni* capsule and *O*-methyl phosphoramidate mutants

**DOI:** 10.1186/s13567-017-0501-y

**Published:** 2018-01-09

**Authors:** Sungwon Kim, Andrea Vela, Sara M. Clohisey, Spiridoula Athanasiadou, Pete Kaiser, Mark P. Stevens, Lonneke Vervelde

**Affiliations:** 1 The Roslin Institute and Royal (Dick) School of Veterinary Studies, University of Edinburgh, Easter Bush, Midlothian, EH25 9RG UK; 2 Scotland’s Rural College, West Main Road, Edinburgh, EH9 3JG UK

## Abstract

*Campylobacter jejuni* is the leading cause of bacterial food-borne gastroenteritis worldwide and human infections are frequently associated with handling and consumption of contaminated poultry. The polysaccharide capsule of *C. jejuni* plays important roles in colonisation of the chicken gut, invasion of epithelial cells and serum resistance and is subject to modification with *O*-methyl phosphoramidate (MeOPN) in most strains. In this study, the cytokine responses of mouse bone marrow-derived macrophages (mBMMs), chicken bone marrow-derived macrophages (chBMMs) and human monocyte-derived macrophages (hMDMs) were measured following infection with *C. jejuni* 11168H wild-type (WT) or isogenic mutants lacking either the capsule (*Δcj1439*) or its MeOPN modification (*Δcj1417*). Consistent with previous observations using murine bone marrow-derived dendritic cells, mutants lacking the capsule or MeOPN elicited enhanced transcription of IL-6 and IL-10 in mBMMs compared to wild-type *C. jejuni*. However, the lack of capsule and MeOPN did not alter IL-6 and IL-10 expression in chBMMs and hMDMs compared to *C. jejuni* WT. Phagocytosis assays showed the acapsular mutant was not impaired in uptake or net intracellular survival after phagocytosis in both chicken and human macrophages; however, the phagocytosis of the MeOPN mutant was significantly decreased in both chicken and human macrophages. In conclusion, differences in the response of macrophages of varying host origin to *Campylobacter* were detected. The absence of MeOPN modification on the capsule of *C. jejuni* did not alter the levels of innate cytokine expression in both chicken and human macrophages compared to the 11168H WT, but affected phagocytosis by host macrophages.

**Electronic supplementary material:**

The online version of this article (10.1186/s13567-017-0501-y) contains supplementary material, which is available to authorized users.

## Introduction

*Campylobacter jejuni* is the leading cause of bacterial food-borne gastroenteritis worldwide [1]. Approximately 70 353 laboratory-confirmed cases of human infection were recorded by national surveillance in the United Kingdom from February 2014 to February 2015 (Zoonoses summary report UK 2014), however the true burden of infection in the community is expected to be far higher [2]. Symptoms of infection in humans vary from mild watery diarrhoea to severe bloody diarrhoea. Disease is usually self-limiting and symptoms last for 5–7 days, but in some cases the infection may be complicated by severe sequelae [3, 4]. Complications of infection include septicaemia and inflammatory neuropathies such as Guillain–Barré syndrome that affect approximately 1/1000 cases [5, 6].

The human immune response to *C. jejuni*, including the role of pattern recognition receptors and bacterial agonists of innate and adaptive immunity, remains poorly understood. As in other enteric infections, innate immune cells are likely to be involved in the initial response to *C. jejuni* infection [7, 8]. Upon *C. jejuni* infection, human monocytic cell line THP-1 and monocyte-derived dendritic cells (DCs) secrete a broad range of cytokines including IL-1β, IL-8, IL-10, IFN-γ, and TNF-α [9, 10]. Additionally, live *C. jejuni* induces a higher cytokine response in DCs than killed bacteria [11]. The pathogen recognition receptors, toll-like receptors (TLR) 2 and 4 have been shown to be involved in signalling and cytokine expression upon infection with *C. jejuni* in vitro using knock-out or knock-down technology [12, 13]. A luciferase-based TLR reporter assay, however, indicated that live *C. jejuni* did not directly activate human TLR2, TLR4, and TLR5 [14]. Human monocytes/macrophages also efficiently kill *C. jejuni* [15].

Chickens are the key reservoir of human *Campylobacter* infection and up to 50–70% of human cases have been attributed to the handling and consumption of contaminated broiler meat [3, 4]. *Campylobacter* colonization of chickens is widespread and results in long-term persistence and shedding [16, 17]. Although large numbers of bacteria are in close contact with the epithelial cell layer, primarily in the large blind caeca (where *C. jejuni* number may exceed 10^9^ CFU/g), no haemorrhagic lesions, caecal dilation and clinical signs of intestines are seen in most colonised birds [18] in clear contrast to humans who develop severe clinical symptoms of diarrhoea. In terms of immune responses, several reports have shown induction of immune-associated gene expression after *Campylobacter* colonization of chickens. Indeed, evidence exists that *C. jejuni* can elicit gut pathology, pro-inflammatory responses and impair weight gain in some, but not all, commercial broiler lines [19]. Experimental infection of a commercial broiler line A1 elicits expression of the pro-inflammatory chemokines CXCLi1, CXCLi2 and IL-1β in the ceca at 2 and 5 days post-infection. Moreover, the infected A1 birds developed diarrhoea at 12 days post-infection [19]. Another study reported that experimental infection of a commercial broiler line (Ross 308) with *C. jejuni* leads to increased mRNA levels of TLR4 and TLR21 in peripheral blood leukocytes, but decreased transcription of other antimicrobial peptide genes in response to *C. jejuni* infection [20]. Additionally, *C. jejuni* infection increases the expression of pro-inflammatory cytokines (IL-1β and IL-6) and chemokines (CXCLi1 and CXCLi2) both in vitro as well as in vivo in ileum and caecum [21–23]. Infection of *C. jejuni* leads to an increased number of circulating monocytes/macrophages in commercial broiler chickens (Ross 308) [20], and an increased number of heterophils are recruited in the ileum and caeca of specific pathogen-free chickens challenged with *C. jejuni* [23]. Similar to human, chicken peritoneal macrophages are able to ingest and kill *C. jejuni* effectively in the in vitro assay [24].

The analysis of the genome sequence of *C. jejuni* NCTC 11168 revealed that approximately 8% of its predicted coding sequences are devoted to the biosynthesis of sugar moieties and bacterial surface glycans including lipo-oligosaccharide (LOS), capsular polysaccharide (CPS) and N- and O-linked glycosylation systems [25]. The CPS plays important roles for both serum resistance and invasion of epithelial cells [26]. The CPS produced by *C. jejuni* strains is structurally complex and highly variable, due in part to the addition of phase-variable modifications such as *O*-methyl, ethanolamine, aminoglycerol and *O*-methyl phosphoramidate (MeOPN) groups [27–29]. Earlier studies reported that approximately 70% of *C. jejuni* isolates have MeOPN modification, which has important roles in invasion of epithelial cells and serum resistance [26, 30]. The loss of MeOPN modification leads to increased invasion of epithelial cells compared to the wild-type (WT) strain but with no effect on adherence [30]. A capsule-deficient mutant (Δ*cj1439*) showed significantly decreased adherence and invasion to epithelial cells compared to *C. jejuni* WT [30, 31]. While the capsule plays a key role in colonisation of the chicken gut [32], the absence of MeOPN modification reportedly does not affect colonisation of *C. jejuni* in the chicken [20], albeit modest attenuation of a MeOPN mutant was observed in piglets [30]. In mouse bone marrow-derived dendritic cells (mBMDCs), both capsule (Δ*cj1439*) and MeOPN (Δc*j1417*) mutants significantly enhances IL-6 and IL-10 mRNA and protein expression compared to WT of *C. jejuni* [33].

In this study, the effect of the capsule and its MeOPN modification on the cytokine responses of macrophages from mice, chickens and human to *C. jejuni* was examined. Both capsule (Δ*cj1439*) and MeOPN (Δ*cj1417*) mutant strains did not alter cytokine responses in chicken and human macrophages, whereas enhanced cytokine responses were observed in mBMMs by the capsule and MeOPN deficient mutants. Furthermore, the lack of MeOPN modification reduced bacterial uptake, but not net intracellular survival, in chicken and human macrophages compared to *C. jejuni* WT.

## Materials and methods

### Animals

Commercial Novogen brown layers and C57/B16 mice were housed in premises licensed under a UK Home Office Establishment License in full compliance with the Animals (Scientific Procedures) Act 1986 and the Code of Practice for Housing and Care of Animals Bred, Supplied or Used for Scientific Purposes. Requests for animals were approved by the local Animal Welfare and Ethical Review Board and animals were humanely culled in accordance with Schedule 1 of the Animals (Scientific Procedures) Act 1986.

### Isolation and culture of mouse bone marrow-derived macrophages (mBMMs)

Vials of frozen C57BL/6 mouse bone marrow were kindly provided by Dr Hu (The Roslin Institute), following recovery by standard methods. The mBMMs were differentiated following the protocol of Irvine et al. [34] in the presence of rhCSF1. Briefly, the bone marrows were cultured on 90-mm plastic petri dishes with RPMI 1640 (Sigma) supplemented with 10% (v/v) FBS (Gibco), 2 mM l-glutamine, 100 U/mL Penicillin, 100 µg/mL Streptomycin (Gibco) and recombinant human colony-stimulating factor-1 (rhCSF1, a gift from Chiron, Emeryville, CA, USA, 1 × 10^4^ U/mL as final concentration) for 7 days for differentiation into macrophages [34]. At day 4 post-seeding, 10 mL of fresh culture media with 10^4^ U/mL rhCSF1 was added per petri dish. At 7 days post-seeding, the differentiated mBMMs were harvested using 10 mM EDTA, and re-plated at a concentration of 1 × 10^6^ cells/well in 6-well plates for inoculation with bacterial strains or agonists and analysis of gene expression. The cells were cultured for 24 h in a humidified 5% CO_2_ atmosphere prior to stimulation.

### Isolation and culture of chicken bone marrow-derived macrophages (chBMMs)

Chicken macrophages were differentiated from bone marrow isolated from embryos at day 20 of development. Bone marrow was harvested by flushing femurs and tibias, and washed once with chilled RPMI 1640 medium. Approximately 1.0 × 10^7^ bone marrow cells were cultured on 90-mm plastic petri dishes as previously described [35] in RPMI 1640 medium supplemented with 10% (v/v) FBS, 10 U/mL Penicillin, 10 μg/mL Streptomycin, 2 mM l-glutamine and 350 ng/mL recombinant chicken CSF1 (rchCSF1) at 41 °C in a humidified 5% CO_2_ atmosphere for 7 days to allow macrophage differentiation. The media was changed on 2 and 4 days post-seeding. At 7 days post-seeding, the differentiated macrophages were harvested using 30 mM EDTA, and re-plated at a concentration of 2 × 10^6^ cells/well in 6-well plates and 5 × 10^5^ cells/well in 24-well plates for gene expression studies and phagocytosis assays, respectively. The cells were cultured for 24 h prior to stimulation.

### Isolation and culture of human CD14^+^ monocyte-derived macrophages (huMDMs)

Human CD14^+^ mononuclear cells were isolated from blood of consenting donors under ethical approval from Lothian Research Ethics Committee (11/AL/0168). Briefly, the buffy coat was isolated from whole blood by centrifugation, followed by isolation of mononuclear cells by gradient separation using Lymphoprep (Stemcell Technologies). The isolated mononuclear cells were washed, and CD14^+^ monocytes were positively selected and purified using MACS with CD14 microbeads (10 µL per 10^7^ cells; Miltenyl Biotec). The purified CD14^+^ monocytes were directly seeded and differentiated on either 6-well plates with 1 × 10^6^ cells/well for gene expression studies or 24-well plates with 5 × 10^5^ cells/well for phagocytosis study, and cultured with RPMI 1640 (Sigma) supplemented with 10% (v/v) FBS (GE Healthcare), 2 mM l-glutamine, 100 U/mL penicillin, 100 µg/mL streptomycin (Sigma), and rhCSF1 (1 × 10^4^ U/mL as final concentration) [34] for 7 days for differentiation into macrophages and achieved a comparable density at the time of inoculation to the avian and murine macrophage cultures. Infections were performed on day 8.

### Bacterial strains, culture and determination of bacteria concentration

Bacterial strains used in this study are *C. jejuni* 11168H (WT), an isogenic Δ*cj1417* mutant lacking phosphoramidate side branch (MeOPN) [36], and acapsular Δ*cj1439* mutant [37] (kindly provided by Prof. Brendan Wren, London School of Hygiene & Tropical Medicine). All bacteria were cultured under microaerophilic conditions (5% O_2_, 5% CO_2_, and 90% N_2_) at 41 °C as described previously [38]. Initially, cultures were grown on modified charcoal-cephoperazone-deoxycholate agar (mCCDA; Oxoid) plates for 48 h, supplemented with selective supplement containing with cefoperazone (32 mg/L) and amphotericin B (10 mg/L; Oxoid), in the presence of kanamycin (50 µg/mL as final concentration) for mutant strains. A single colony from the initial culture was selected and inoculated to 10 mL of Mueller–Hinton broth (Oxoid), followed by additional culture for 24 h at 41 °C with 400 rpm shaking. Twenty-four hours post-inoculation, morphology and motility of the bacteria was observed using KOVA Glasstic^®^ slide with grid (Kova), with only spiral-shaped and rapidly motile cultures being used. Absorbance was measured at 600 nm wavelength. For live bacteria, the cultured bacteria were collected by centrifugation at 13 000 × *g* for 10 min. The bacterial pellets were washed with sterile PBS once, and then resuspended with PBS. The number of live bacteria used for stimulation was subsequently verified by serial dilution and plating to establish viable bacterial cell count.

To fix the bacteria, the cultured bacteria were pelleted by centrifugation at 13 000 × *g* for 10 min. The bacteria were resuspended in 200 μL of sterile PBS, and mixed with an equal volume of 4% (w/v) paraformaldehyde in PBS for 1 h at room temperature. The bacteria were washed 3 times with PBS prior to incubation with macrophages. Bacterial fixation was confirmed by microscopic observation and plating to establish elimination of viability.

### Stimulation of macrophages with *C. jejuni*

The chBMMs and mBMMs were harvested and re-plated in 6-well plates 24 h prior to stimulation. The live and fixed bacteria were diluted with RPMI 1640 medium supplemented with 10% (v/v) FBS, 2 mM l-glutamine and either rchCSF1 or rhCSF1, and were added to the prepared cells at MOIs of 50 or 100 for 3 or 6 h for subsequent analysis. For huMDMs, diluted bacteria were added directly into the huMDMs differentiated for 8 days. Lipopolysaccharide from *Salmonella minnesota* R595 (100 ng/mL as final concentration; InvivoGen) [39] was used as a positive control and PBS in the culture media as negative control.

### Quantitative real-time PCR (qRT-PCR)

Total RNA was extracted from the stimulated macrophages using the RNeasy Mini Spin Column (Qiagen) and 1 μg of total RNA was reverse transcribed with cDNA synthesis kit (Bio-Rad), followed by 1:5 dilution for target transcripts and 1:100 dilution for 28S ribosomal RNA. To measure mRNA level from the chBMMs, 2 μL of diluted cDNA was used with TaqMan^®^ Fast Universal PCR Master Mix (Applied Biosystems, UK) and TaqMan primer and probes (Table 1). For the mRNA levels of human or mouse macrophages, 2 μL of diluted cDNA was mixed with Fast SYBR^®^ Green Master Mix (Applied Biosystems) and specific primer sets (Table 1). All qRT-PCR was performed by Applied Biosystems 7500 Fast real-time PCR system. Cycle thresholds were normalised to internal control (28 s for chicken and human and β-actin for mouse) and calibrated to a medium-treated control sample for relative quantification using the ΔΔCt method [40]. The results were presented as a fold change.

**Table 1:** **Primer sequences for qRT-PCR analyses of cytokine transcripts**

Gene	Forward primer	Probe	Reverse primer	Accession no.
Chicken				
28S	GGCGAAGCCAGAGGAAACT	AGGACCGCTACGGACCTCCACCA	GACGACCGATTTGCACGTC	AH001604
IL-1β	GCTCTACATGTCGTGTGTGATGAG	CCACACTGCAGCTGGAGGAAGCC	TGTCGATGTCCCGCATGA	NM_204524
IL-6	GCTCGCCGGCTTCGA	AGGAGAAATGCCTGACGAAGCTCTCCA	GGTAGGTCTGAAAGGCGAACAG	NM_204628
CXCLi1	TGGCTCTTCTCCTGATCTCAATG	TCGCTGAACGTGCTTGAGCCATACCTT	GCACTGGCATCGGAGTTCA	Y14971
IL-10	CATGCTGCTGGGCCTGAA	CGACGATTCGGCGCTGTCACC	CGTCTCCTTGATCTGCTTGATG	NM_001004414
Human				
28S	GAATTCATGGACGACACGAG		ACTGTGACAGACCATTCCCA	NR_003279
IL-6	CCAGAGCTGTGCAGATGAGT		CTGCGCAGAATGAGATGAGT	NM_000600
IL-10	GAGGCTACGGCGCTGTCAT		TGCTCCACGGCCTTGCT	NM_000572
Mouse				
β-actin	TCCAGCCTTCCTTCTTGGGT		GCACTGTGTTGGCATAGAGGT	NM_007393
IL-6	GGGACTGATGCTGGTGACAACC		GGAGAGCATTGGAAATTGGG	NM_031168
IL-10	CGATGACGGGCCAGTGAGAATG		TCAACACGTGGGCAGGATAGGCT	NM_010548

### Phagocytosis assay

The assay was performed as described previously [41], with slight modifications. Twenty-four hours prior to stimulation, 5 × 10^5^ cells/well of chBMMs were seeded into 24-well plates. For huMDMs, the purified CD14^+^ monocytes were seeded on 24-well plates at a density of 5 × 10^5^ cells/well. The prepared cells were inoculated at an MOI of 50 for 30 min without antibiotics. Then, the cells were washed three times with PBS, followed by treatment with gentamicin (200 µg/mL as a final concentration) for 30 min to kill extracellular bacteria. The cells were washed three times with PBS, incubated for 0 or 3 h with RPMI 1640 supplemented with 10% (v/v) FBS, 20 µg/mL of gentamicin and either rchCSF1 or rhCSF1, and subsequently lysed with 0.5% Triton X-100 on ice. The collected lysates were prepared by tenfold serial dilution with PBS and plated on mCCDA plates to determine intracellular bacteria. The colonies were counted, followed by the calculation of the CFU/mL and the percentage of killed bacteria to measure net intracellular survival.

### Statistical analysis

All data were analysed by Student’s *t* test or one-way analysis of variance (ANOVA) using program R [42], and significant differences between groups were considered significant by Tukey’s honest significant difference (HSD) test at *P* < 0.05 (confidence level = 95%).

## Results

### Capsule and MeOPN mutants of *C. jejuni* elicit enhanced cytokine responses in mBMMs

Rose et al. [33] previously described the induction of differential responses in murine dendritic cells using the same strains of *C. jejuni*. To ensure that the bacteria used in our study would give similar responses, we repeated the study of Rose and stimulated mBMDCs at a multiplicity of infection (MOI) of 50 with live or fixed wild-type (WT) *C. jejuni* 11168H or isogenic capsule (Δ*cj1439*) and MeOPN (Δ*cj1417*) mutants for 3 h. We confirmed that mBMDCs expressed significantly enhanced IL-6 and IL-10 mRNA levels after stimulation with the capsule and MeOPN deficient mutants of *C. jejuni* (see Additional file 1).

**Figure 1:**
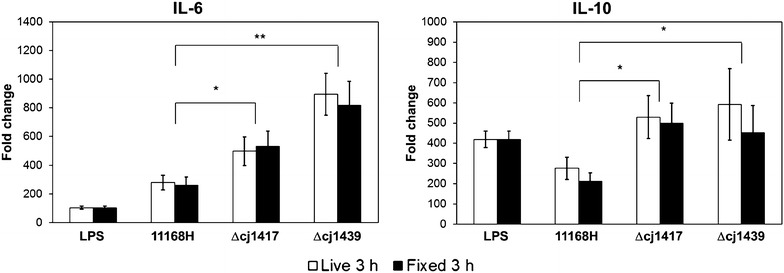
***Campylobacter jejuni*** **capsule and MeOPN mutants elicit an enhanced cytokine responses in mBMMs.** Live or fixed *C. jejuni* 11168H (WT), Δ*cj1417* (MeOPN) and Δ*cj1439* (capsule) mutants were incubated with mBMMs at MOI 50 for 3 h. *Salmonella* LPS was used as a positive control, while PBS in the medium was used as a negative control. Graphs show representative results of two independent experiments and error bars are the standard deviation of two independent experiments. Asterisks indicate a statistically significant difference of mRNA levels of inflammatory-related cytokines compared to *C. jejuni* WT (*P < 0.05; **P < 0.01).

After confirming that the bacteria induced similar responses to previously published data [33], we then stimulated mBMMs with the same strains of *C. jejuni* under the same condition to examine the effect of capsular and its modification on macrophage responses. mBMMs expressed significantly higher mRNA levels of IL-6 and IL-10 after stimulation with the capsule and MeOPN deficient mutants of *C. jejuni* compared to the *C. jejuni* WT (Figure 1). The expression levels of the cytokines did not differ after stimulation with live or fixed *C. jejuni*.

### Cytokine responses of chBMMs and huMDMs to *C. jejuni* are not affected by the capsule of MeOPN modification

**Figure 2:**
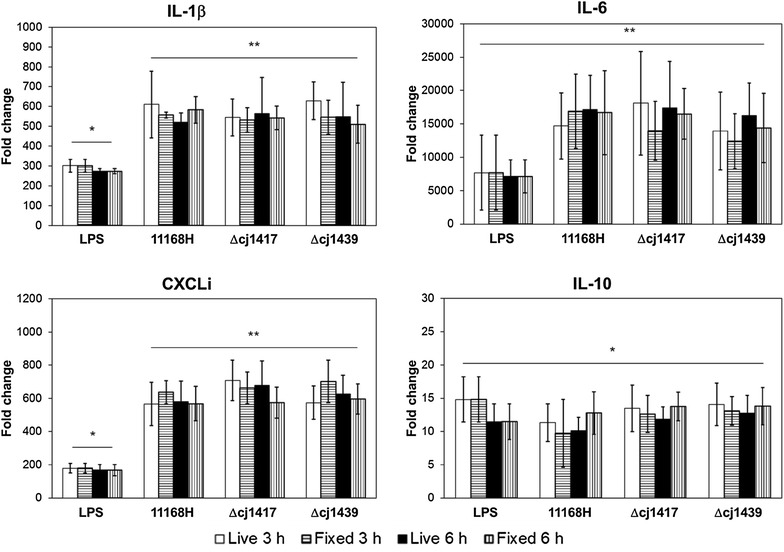
**Wild-type and capsule or MeOPN deficient strains of** ***C. jejuni*** **induce similar cytokine responses in chBMMs.** Live or fixed *C. jejuni* 11168H (WT), Δ*cj1417* (MeOPN) and Δ*cj1439* (capsule) mutants were incubated with chBMMs at MOI 50 for 3 and 6 h. *Salmonella* LPS (100 ng/mL) was used as a positive control and mRNA levels of target genes were presented as folder changes compared to PBS in the medium (a negative control). Graphs show representative results of three independent experiments and error bars are standard deviation of three independent experiments. Asterisks indicate a statistically significant difference of mRNA levels of inflammatory-related cytokines compared to PBS in the medium (*P < 0.01; **P < 0.001).

To examine the role of the capsule and its MeOPN modification on the innate immune response of chicken macrophages to *C. jejuni*, live or fixed *C. jejuni* WT and capsule (Δ*cj1439*) and MeOPN (Δ*cj1417*) mutants were incubated with chBMMs for 3 and 6 h at an MOI of 50, and transcripts of inflammation-related signature cytokines were measured (Figure 2). The *C. jejuni* WT, capsule (Δ*cj1439*) and MeOPN (Δ*cj1417*) mutants all significantly induced transcription of the pro-inflammatory cytokines IL-1β and IL-6, the chemokine CXCLi1, and anti-inflammatory cytokine IL-10, compared to the negative control incubated with PBS (Figure 2). A similar induction of responses was observed with live or fixed bacterial cells (Figure 2). However, in contrast to observations with murine cells, no statistically significant differences in the mRNA levels of IL-1β, IL-6, CXCLi1 and IL-10 were detected after stimulation with the WT or mutant strains of *C. jejuni*. Increasing the MOI to 100 did not result in significantly different transcript levels for the cytokines, either across the strains or time intervals measured (see Additional file 2).

Since TLR4 can trigger inflammatory cytokine response in mammalian innate immune cells by binding to LOS of *C. jejuni* [10, 43], changes in chicken TLR4 transcript levels upon the stimulation with WT and mutant *C. jejuni* strains were investigated (see Additional file 3). Stimulation of chBMMs with *Salmonella* LPS for 3 h led to the downregulation of TLR4 mRNA compared to the cells incubated with medium, as previously reported [44, 45]. Expression of TLR4 mRNA was significantly decreased following stimulation with all three strains of *C. jejuni*; however there was no difference of chicken TLR4 transcript levels observed among WT, capsule (Δ*cj1439*) and MeOPN (Δ*cj1417*) mutants of *C. jejuni* regardless of whether live or fixed bacterial cells were used (see Additional file 3).

**Figure 3:**
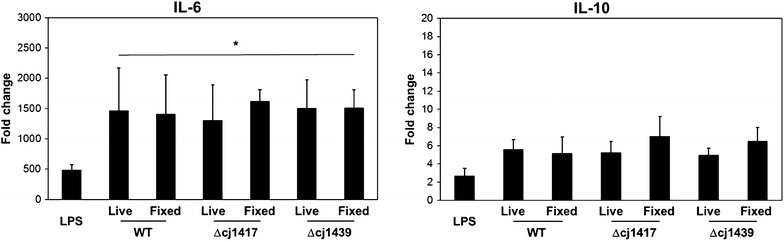
**Wild-type and capsule or MeOPN deficient strains** ***C. jejuni*** **induce similar cytokine responses in huMDMs**. Live or fixed *C. jejuni* 11168H (WT), Δ*cj1417* (MeOPN) and Δ*cj1439* (capsule) mutants were incubated with huMDMs at MOI 50 for 3 h. *Salmonella* LPS was used as a positive control, and mRNA levels of target gene was presented as folder changes compared to PBS in the medium (a negative control). Graphs show representative results of three individual donors (two individuals for fixed *C. jejuni*). Error bars are standard deviation of three (two) independent experiments. Asterisks indicate a statistically significant difference of mRNA levels for the indicated inflammation-related cytokines compared to PBS in the medium (*P < 0.01).

Since *C. jejuni* is a zoonotic bacterium with relatively contrasting symptoms in chicken and human, human CD14^+^ MDMs were cultured and stimulated with an MOI 50 of live or fixed WT or mutant *C. jejuni* for 3 h to compare inflammatory responses with macrophages or avian and murine origin (Figure 3). All three strains of *C. jejuni* elicited expression of IL-6 mRNA in huMDMs regardless of the bacteria being live or fixed, but no significant differences between WT and capsule (Δ*cj1439*) or MeOPN (Δ*cj1417*) mutants were found. Unlike IL-6, IL-10 transcription in huMDM were not affected by neither *Salmonella* LPS nor the different strains of *C. jejuni*.

### Reduced number of intracellular *C. jejuni* in chicken and human macrophages due to the lack of MeOPN modification

**Figure 4:**
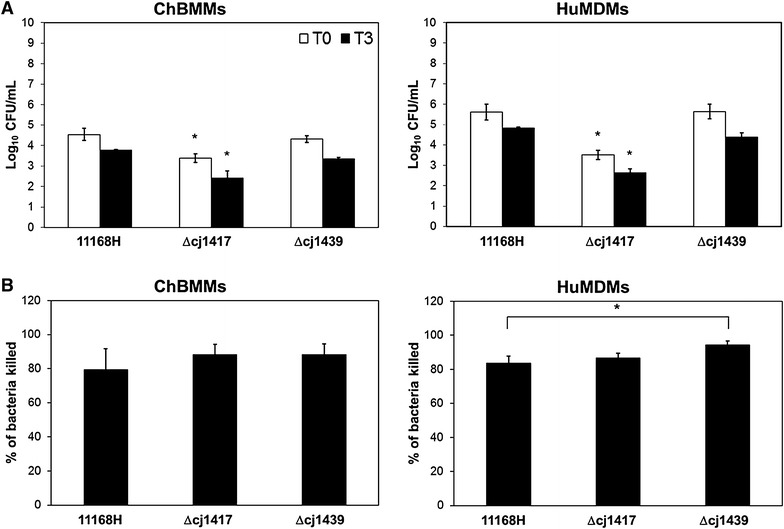
**The lack of MeOPN modification affects the number of intracellular** ***C. jejuni*** **in macrophages.** Chicken or human macrophages were incubated with *C. jejuni* 11168H (WT), Δ*cj1417* (MeOPN) and Δ*cj1439* (capsule) mutant at MOI 50 for 30 min. Intracellular bacteria were assessed by a gentamicin protection assay, and counted at 0 h after removal of gentamicin (T0, open bar) and after a further 3 h (T3, closed bar) of incubation. The average number of colonies at T0 and T3 are presented as Log_10_ CFU/mL (**A**). The percentage of killed bacteria was calculated using the average number of colonies at T0 and T3 (**B**). Each bar represents mean of three independent experiments and error bars are the standard deviation of three independent experiments. Asterisks indicate a statistically significant difference of the number of survived bacteria (**A**) and the percentage of killed bacteria (**B**) in different treatment (*P* < 0.05).

To investigate the effect of the capsule and MeOPN modification on *C. jejuni* uptake and net intracellular survival in both chicken and human macrophages, a phagocytosis assay was performed (Figure 4). Bacterial recoveries, after gentamicin treatment to kill extracellular bacteria, were used to quantify initial phagocytosis (presented as 0 h) and net intracellular survival (3 h after removal of gentamicin). In both chBMMs and huMDMs, there was no statistically significant differences in the number of *C. jejuni* taken up (T0) between the wild-type strain and acapsular mutant in macrophages. However, the lack of MeOPN modification led to a significantly reduced uptake (*P* < 0.03) of intracellular *C. jejuni* in chBMMs and huMDMs compared to the WT and acapsular mutant (Figure 4A). The percentage of bacteria surviving at 3 h post-incubation revealed no significant difference of net intracellular survival between WT and MeOPN mutants in chBMMs and huMDMs (Figure 4B). The acapsular mutant showed a slight but significant increase in the percentage of bacteria killed in huMDMs, but not chBMMs, 3 h post-incubation compared to WT of *C. jejuni*.

## Discussion

There has been concerted effort to better understand the differences between the avian and human immune response to *C. jejuni* infection [9, 10, 14, 21]. Recently, important roles for the capsule and its MeOPN modification have been demonstrated in cell adherence, invasion, colonisation, serum resistance and host immune responses [26, 30, 31, 33]. In this study, we compared the cytokine responses of macrophages of different host origin known to differ in their symptoms upon infection with *C. jejuni*. The phagocytic and killing capacity of chicken and human macrophages to *C. jejuni* and capsule or MeOPN deficient mutants were analysed to better understand these differential responses observed in vivo. Although macrophages are not the first cells to contact *C. jejuni* during the infection in mucosal environment, intestinal and colonic macrophages are part of the first line defence mechanism since they are located strategically underneath the epithelial lining in close proximity to large numbers of luminal bacteria and antigenic stimuli. These gut macrophages regulate inflammatory responses to bacteria and antigens that cross the epithelium, protect the mucosa against harmful pathogen and scavenge dead cells and foreign debris by crosstalking with adjacent intestinal epithelial cells through TLR-dependent signalling pathway [46, 47]. Additionally, a recent study reported crosstalk between intestinal epithelial cells and macrophages mediated through TLR4, resulting in the production of IL-10 from human intestinal epithelial cells [48].

Using the current WT, capsule (Δ*cj1439*) and MeOPN (Δ*cj1417*) strains of *C. jejuni*, we were able to reproduce the enhanced expression of IL-6 and IL-10 mRNA levels in mBMDCs by capsule and MeOPN deficient strains of *C. jejuni* compared to the WT strain, as reported by Rose et al. [33]. We also found that mBMMs incubated with the mutant strains expressed elevated levels of IL-6 and IL-10 mRNA compared to the *C. jejuni* WT, suggesting a potential role for capsule and MeOPN modification in bacterial evasion of mouse innate immune responses. In the context of zoonosis however, it is more relevant to consider the response of avian and human cells to *C. jejuni* infection and the role of bacterial factors in this process. For this purpose, the mRNA levels of IL-1β, IL-6 and CXCLi1 (pro-inflammatory cytokine/chemokine) and IL-10 (anti-inflammatory cytokine) were measured to examine the immune response of chicken, based on Smith et al. [21]. As markers of human responses, the expression of IL-6 and IL-10 mRNA was measured based on Rose et al. [33]. All three strains of *C. jejuni* significantly induced mRNA levels of inflammation-related signature genes in both chicken and human macrophages, indicating that *C. jejuni* is highly immunogenic to both avian and human primary macrophages. These results are very similar with early reports that *C. jejuni* is highly immunogenic to both human and mouse macrophage cell lines [14, 20]. However, neither capsule nor MeOPN deficient mutants altered the cytokine responses in chicken and human macrophages, when compared to WT, in contrast to the responses of mouse macrophages, indicating that neither of structures is vital for the induction of an immune response in human and avian cells.

All three strains of *C. jejuni* induced significant mRNA levels of IL-10 in chBMMs and mBMMs compared to PBS in the medium (as negative control). However, there was no significant increase in IL-10 transcripts in huMDMs observed by *C. jejuni* WT, capsule (Δ*cj1439*) and MeOPN (Δ*cj1417*) mutants. In fact, the expression levels of IL-10 mRNA in huMDMs from all three donors was relatively high compared to chBMMs and mBMMs, although the high expression of IL-10 mRNA did not appear to affect the expression of IL-6 during the stimulation with *C. jejuni*. Except for the different origin of macrophages between bone marrow and monocytes, there is no obvious explanation for the different responses of IL-10 mRNA levels in huMDMs to *C. jejuni* compared to chBMMs and mBMMs.

It has been reported that the capsule (Δ*cj1439*) mutant showed significantly decreased adherence and invasion to the epithelial cells [30, 31], while the MeOPN (Δ*cj1417*) mutant has shown increased invasion (but no defect in adherence) to epithelial cells compared to wild-type strains [30]. Additionally, the lack of MeOPN modification, as well as capsule, leads to the significantly decreased serum resistance, indicating a potentially protective role for MeOPN modification against the innate immune system [30]. Our phagocytosis study showed decreased *C. jejuni* uptake in the absence of MeOPN modification in chicken and human macrophages compared to *C. jejuni* WT; however, the lack of MeOPN modification did not affect net intracellular survival in chicken and human macrophages. On the other hand, the capsule did not affect bacterial uptake or net intracellular survival at least at the time intervals sampled.

In summary, chicken and human macrophages showed similar responses to *C. jejuni* WT, capsule (Δ*cj1439*) and MeOPN (Δ*cj1417*), unlike mouse macrophages. The absence of MeOPN modification on the capsule of *C. jejuni* did not alter the cytokine responses, as well as net intracellular survival in chicken and human macrophages compared to *C. jejuni* 11168H, but it affected *C. jejuni* uptake by chicken and human macrophages. The study indicates that extrapolation of findings regarding the potential contribution of candidate virulence factors and host responses to pathogenesis may not always be reproduced across comparable cell types originating from other hosts.

## Additional files

**Additional file 1.** ***Campylobacter jejuni*** **capsule and MeOPN mutants elicit an enhanced cytokine responses in mBMDCs.** The mBMDCs were differentiated from vials of frozen C57BL/6 mouse bone marrow following the protocol of Rose et al. [31] in the presence of GM-CSF (final concentration of 20 ng/mL; Peptro Tech) for 7 days. Live or fixed *C. jejuni* 11168H (WT), Δ*cj1417* (MeOPN) and Δ*cj1439* (capsule) mutants were incubated with mBMDCs at MOI 50 for 3 h. *Salmonella* LPS was used as a positive control, while PBS in the medium was used as a negative control. Graphs show representative results of two independent experiments. Error bars are the standard deviation of two independent experiments. Asterisks indicate a statistically significant difference of mRNA levels of inflammatory-related cytokines compared to *C. jejuni* WT (*P < 0.05; **P < 0.01).

**Additional file 2.** **Analysis of cytokine responses of chBMMs when inoculated at an MOI 100 with the** ***C. jejuni*** **WT and capsule or MeOPN deficient strains.** Live or fixed *C. jejuni* 11168H (WT), Δ*cj1417* (MeOPN) and Δ*cj1439* (capsule) mutants were incubated with chBMMs at MOI 100 for 3 and 6 h. *Salmonella* LPS (100 ng/mL) was used as a positive control and PBS in the medium as a negative control. Graphs show representative results of three independent experiments. Error bars are the standard deviation of three independent experiments. Asterisks indicate a statistically significant difference of mRNA levels of inflammation-related cytokines compared to PBS in the medium (*P < 0.01; **P < 0.001).

**Additional file 3.** **Analysis of TLR4 transcription in chBMMs stimulated wild-type** ***C. jejuni*** **and capsule or MeOPN mutants.** Live or fixed *C. jejuni* 11168H (WT), Δ*cj1417* (MeOPN) and Δ*cj1439* (capsule) mutants were incubated with chBMMs at MOI 50 for 3 h. *Salmonella* LPS (100 ng/mL) was used as a positive control and PBS in the medium as a negative control. Graphs show representative results of three independent experiments, and error bars are the standard deviation of three independent experiments. Asterisks indicate a statistically significant difference of TLR4 transcripts compared to PBS in the medium (*P* < 0.05).

## Abbreviations

DCs: dendritic cells
TLR: toll-like receptors
LOS: lipo-oligosaccharide
CPS: capsular polysaccharide
MeOPN: *O*-methyl phosphoramidate
mBMDCs: mouse bone marrow-derived dendritic cells
mBMMs: mouse bone marrow-derived macrophages
chBMMs: chicken bone marrow-derived macrophages
huMDMs: human monocytes-derived macrophages
rhCSF1: recombinant human colony-stimulating factor-1
rchCSF1: recombinant chicken colony-stimulating factor-1
mCCDA: modified charcoal-cephoperazone-deoxycholate agar
MOI: multiplicity of infection
