# Supplementary figures and images for: Host-specific differences in the response of cultured macrophages to Campylobacter jejuni capsule and O-methyl phosphoramidate mutants
Source: Vet Res. 2018 Jan 9;49:3. doi: 10.1186/s13567-017-0501-y (PMC5759256; doi:10.1186/s13567-017-0501-y)

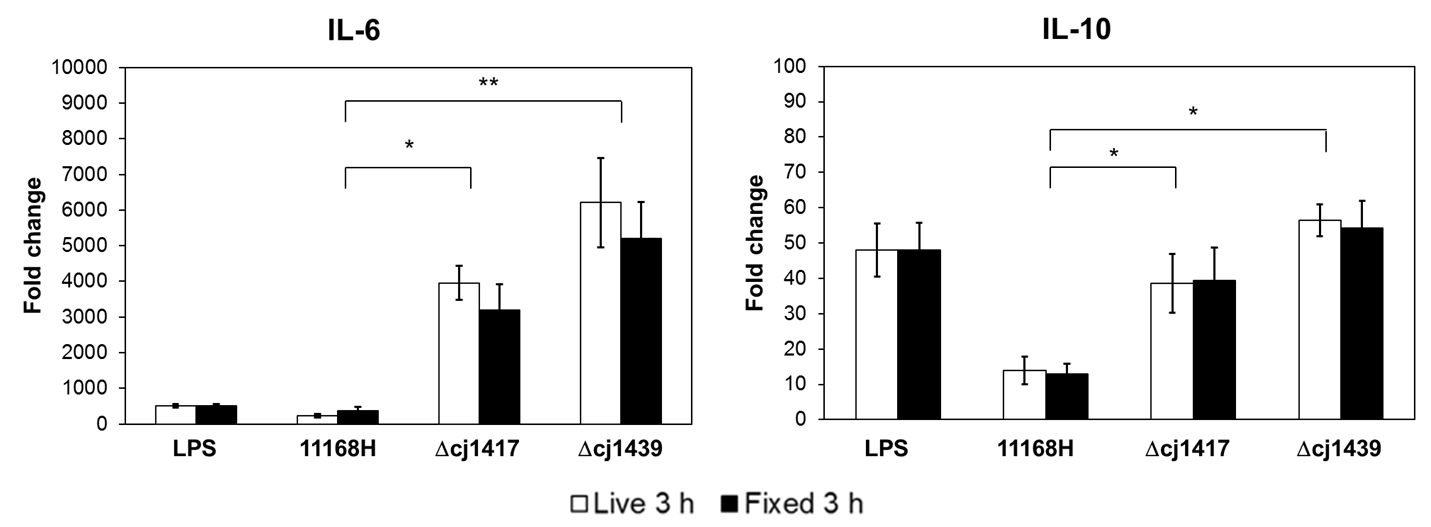

Supplement: Supplementary file 1 — Additional file 1. Campylobacter jejuni capsule and MeOPN mutants elicit an enhanced cytokine responses in mBMDCs. The mBMDCs were differentiated from vials of frozen C57BL/6 mouse bone marrow following the protocol of Rose et al. [31] in the presence of GM-CSF (final concentration of 20 ng/mL; Peptro Tech) for 7 days. Live or fixed C. jejuni 11168H (WT), Δcj1417 (MeOPN) and Δcj1439 (capsule) mutants were incubated with mBMDCs at MOI 50 for 3 h. Salmonella LPS was used as a positive control, while PBS in the medium was used as a negative control. Graphs show representative results of two independent experiments. Error bars are the standard deviation of two independent experiments. Asterisks indicate a statistically significant difference of mRNA levels of inflammatory-related cytokines compared to C. jejuni WT (*P < 0.05; **P < 0.01). [file 13567_2017_501_MOESM1_ESM.docx]

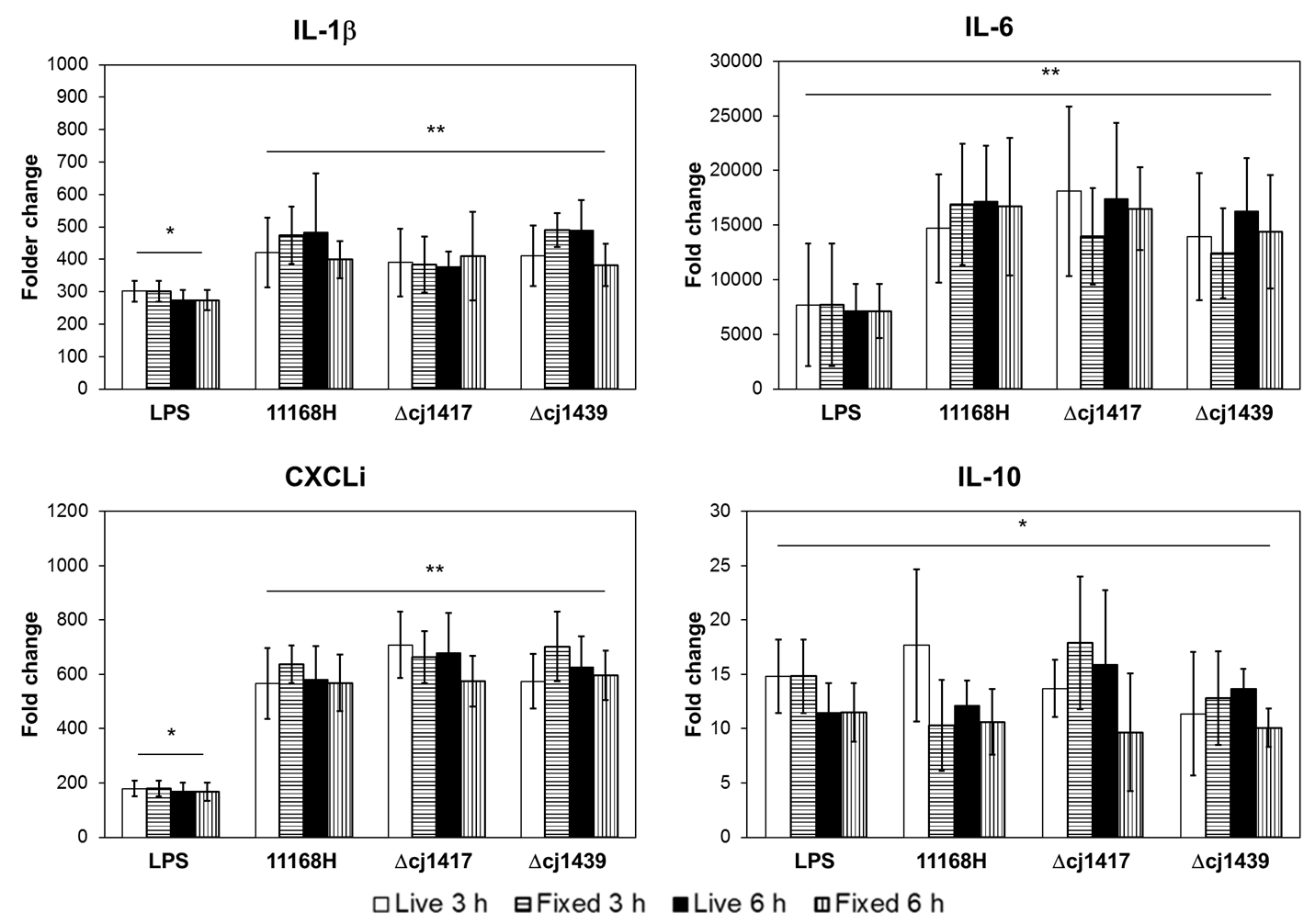

Supplement: Supplementary file 2 — Additional file 2. Analysis of cytokine responses of chBMMs when inoculated at an MOI 100 with the C. jejuni WT and capsule or MeOPN deficient strains. Live or fixed C. jejuni 11168H (WT), Δcj1417 (MeOPN) and Δcj1439 (capsule) mutants were incubated with chBMMs at MOI 100 for 3 and 6 h. Salmonella LPS (100 ng/mL) was used as a positive control and PBS in the medium as a negative control. Graphs show representative results of three independent experiments. Error bars are the standard deviation of three independent experiments. Asterisks indicate a statistically significant difference of mRNA levels of inflammation-related cytokines compared to PBS in the medium (*P < 0.01; **P < 0.001). [file 13567_2017_501_MOESM2_ESM.docx]

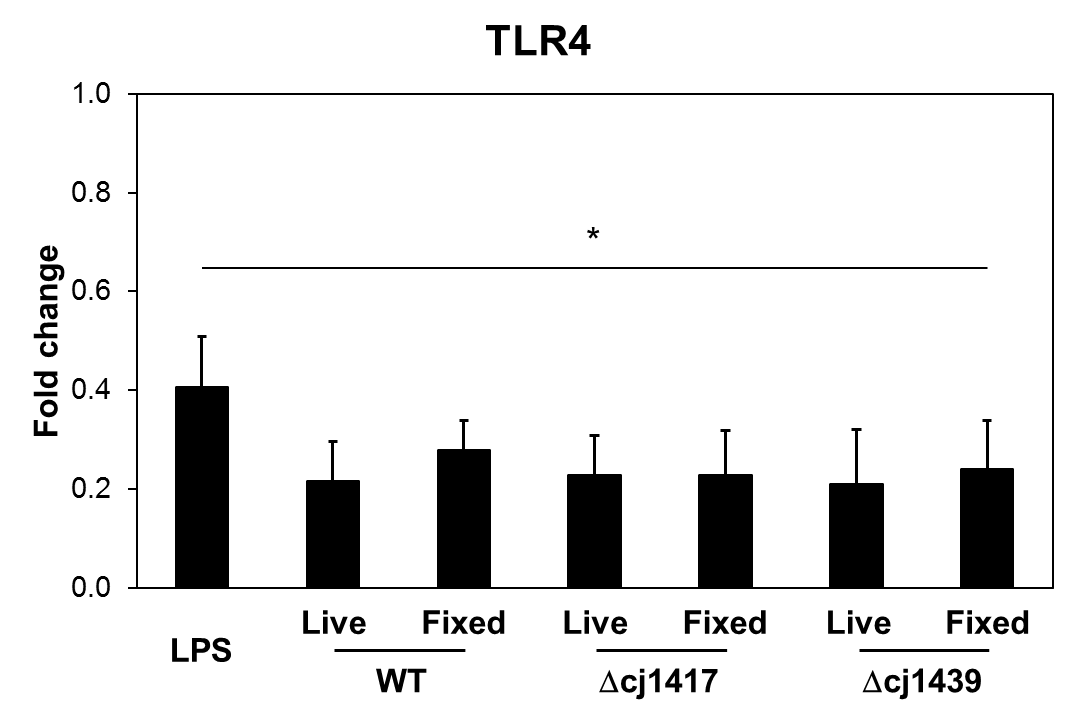

Supplement: Supplementary file 3 — Additional file 3. Analysis of TLR4 transcription in chBMMs stimulated wild-type C. jejuni and capsule or MeOPN mutants. Live or fixed C. jejuni 11168H (WT), Δcj1417 (MeOPN) and Δcj1439 (capsule) mutants were incubated with chBMMs at MOI 50 for 3 h. Salmonella LPS (100 ng/mL) was used as a positive control and PBS in the medium as a negative control. Graphs show representative results of three independent experiments, and error bars are the standard deviation of three independent experiments. Asterisks indicate a statistically significant difference of TLR4 transcripts compared to PBS in the medium (P < 0.05). [file 13567_2017_501_MOESM3_ESM.docx]
